# A triflate and alkynyl protected Ag_43_ nanocluster with a passivated surface†

**DOI:** 10.1039/d0ra01244k

**Published:** 2020-05-20

**Authors:** Ting Li, Xiaoqin Cui, Linfeng Liang, Cui Luo, Huan Li, Xian-Ming Zhang

**Affiliations:** School of Chemistry & Material Science, Shanxi Normal University Linfen 041004 China xmzhang@sxu.edu.cn; Institute of Crystalline Materials, Shanxi University Taiyuan 030006 China 59584340@sxu.edu.cn

## Abstract

A trifluoromethanesulfonate (OTf) and *tert*-butylacetylene (^*t*^BuC≡C^−^) co-protected silver nanocluster (NC), Ag_43_(^*t*^BuC≡C)_24_(CF_3_SO_3_)_8_ (Ag_43_), was synthesized and characterized. Single crystal X-ray diffraction analysis revealed its total structure. 43 Ag atoms are arranged into a three-concentric-shell Ag@Ag_12_@Ag_30_ structure. Both OTf and ^*t*^BuC≡C^−^ ligands bonded with Ag atoms in a μ_3_ mode. The application of Ag_43_ as a catalyst for the reaction of silane with alcohol or H_2_O indicated that the surface ligands had a profound passivation effect, which significantly influenced the reactivity and selectivity.

## Abstract 2

A trifluoromethanesulfonate (OTf) and *tert*-butylacetylene (^*t*^BuC≡C^−^) co-protected silver nanocluster (NC), Ag_43_(^*t*^BuC≡C)_24_(CF_3_SO_3_)_8_ (Ag_43_) was synthesized and characterized. Its surface ligands have shown strong passivation effect in the reaction catalyzed by Ag_43_.

## Graphical abstract

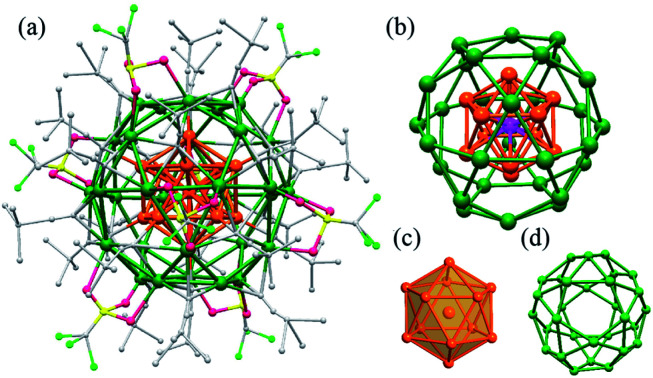

## Introduction

Metal NCs have attracted a great amount of attention during the past decades not only for their aesthetic values but also for their potential applications in self-assembly, surface plasmon resonance (SPR), bio-markers, (electro)catalysis, *etc.*^1–6^ Fundamentally relating their structures with their properties, however, has encountered huge challenges.^7^ One obstacle is the difficulty in precisely characterizing the structures of NCs. To this end, many practical methodologies have been brought forward. Among them, single-crystal crystallography has special advantages in determining the total structures of NCs.^8^ Aiming to resolve the target's structure at atomic level, this methodology has found incomparable advantage in revealing the organic–metal interfacial structure of NCs. For example, the unprecedented “staple” bonding motif of thiolate of Au_102_ NC was firstly revealed by this method.^9,10^ There has since been a boom in the study of the potential applications of the atomically precise NCs, especially catalysis.^11^ The promoting effect of the surface ligands have been repeatedly observed in different types of reactions.^12,13^ However, to obtain the single crystals of NCs, the strong interaction between the NCs and ligands is usually necessary for stabilizing the NCs. Thus, the surface catalytically active sites might be blocked by the organic ligands. However, the researches addressing this issue of Ag NCs seem rare.

On the other side, a large number of thiolate and(or) phosphine protected Au or Ag NCs have been synthesized so far, such as Au_25_,^14^ Au_38_,^15^ Au_279_,^16^ Ag_40_,^17^ Ag_44_,^18,19^ Ag_67_,^20^ Ag_146_,^21^ Ag_141_,^22^ and many others.^23^ Beyond the conventional ligands, alkynes are drawing fast-increasing attentions, thanks to the flexible bonding modes of alkynes with metals.^24–28^ In recent years, there have been a considerable amount of NCs containing anions in their structures. Most of them, however, function as templates or counterions.^29–32^ Ag NCs or complexes featuring direct Ag–O bonding are mostly those with Ag(i) species.^33^ Examples of Ag(0) NCs protected by inorganic oxo anions are rare. Sun *etc.* recently reported a Ag(0) NC co-protected by CrO_4_^2−^ and ^*t*^BuC≡C^−^, [Ag_48_(C≡C^*t*^Bu)_20_(CrO_4_)_7_].^34^ Herein, we report the first example of a Ag(0) NC co-protected by triflate (OTf) and *tert*-butylacetylene (^*t*^BuC≡C^−^), namely, Ag_43_(^*t*^BuC≡C)_24_(CF_3_SO_3_)_8_ (denoted as Ag_43_). The detailed structure of Ag_43_ is analyzed and discussed. Significantly, we have found a strong passivation effect in the reaction of silanes with alcohol or H_2_O catalyzed by Ag_43_. This effect not only sharply decreased the reaction rate, but also exerted a direct influence on the reaction selectivity.

## Results and discussion

**Fig. 1:**
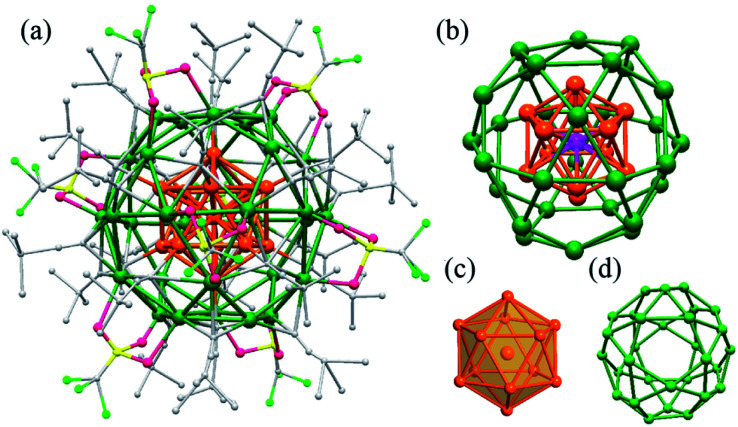
(a) The overall structure of Ag_43_(^*t*^BuC≡C)_24_(CF_3_SO_3_)_8_, Ag_43_. (b) The three shells of Ag@Ag_12_@Ag_30_. (c) The Ag@Ag_12_ centered icosahedron. (d) The outer Ag_30_ shell. Color legend: olive green and orange, Ag; pink, O; yellow, S; light green, F; gray, C. H atoms are omitted for clarity.

Ag_43_ was synthesized by introducing a reducing agent, NaBH_4_ into an ethanol solution of AgSO_3_CF_3_, *tert*-butylacetylene (^*t*^BuC≡C^−^), 1,4-bis-(diphenylphosphino)butane (dppb), and triethylamine (Et_3_N). The reaction was aged for 24 hours during which the colour gradually changed from pale-yellow to dark green. A block-shaped single crystal was obtained by slowly diffusing a mixed solvent of hexane and ether into the mother solution (ESI, Fig. S1†). Ag_43_ crystallizes in *I*2/*m* space group and its overall structure is shown in Fig. 1 and S2.† It consists of 43 silver atoms, 24 ^*t*^BuC≡C^−^ and 8 OTf ligands. The Ag atoms are arranged into a three-shell Russian doll structure, Ag@Ag_12_@Ag_30_ (Fig. 1b). The Ag@Ag_12_ shells make up an icosahedron with a dodeca-coordinated Ag atom at the center (Fig. 1c). Bearing a Ag atom bonding with only Ag atoms endows Ag_43_ with partial Ag(0) character. The rest 30 Ag atoms of the outermost shell form 12 pentagons and 20 triangles. The two regular polygons are connected through edge-sharing, which makes each pentagon be enclosed by five triangles and each triangle by three pentagons. Geometrically, such an arrangement forms an icosidodecahedron, an Archimedean solid (Fig. 1d). The outermost shell is closely related with the ligands' distribution (see below). The average Ag–Ag bond length between the central Ag to Ag_12_ shell is 2.858 Å, while the value is 3.1 Å between Ag_12_ and Ag_30_ shells. Both are less than the sum of the van der Waals radii of two Ag atoms (3.44 Å), indicating argyrophilic interactions between the shells.

**Fig. 2:**
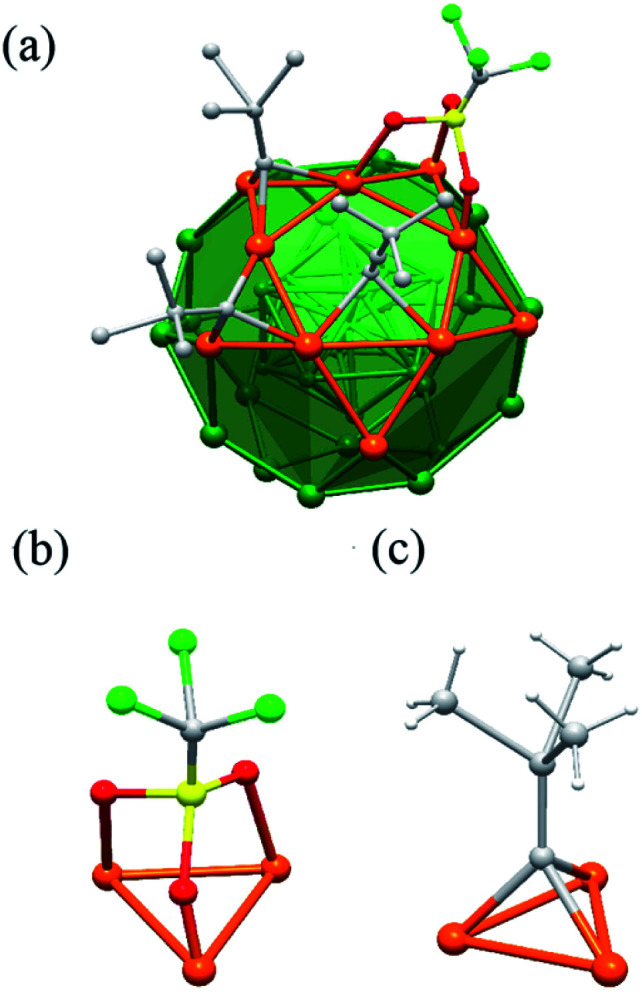
(a) The coordination mode of Ag_43_ surface ligands. The μ_3_ bonding motifs of OTf (b) and ^*t*^BuC≡C^−^ (c) with the Ag_30_ shell. For clarity, only parts of the ligands are shown.

As mentioned, the distribution of peripheral ligands is dependent on the arrangement of silver atoms, especially the Ag_30_ shell (Fig. 2a). There are 24 ^*t*^BuC≡C^−^ ligands which can be equally divided into two groups. Each of the first half penetrates one of the twelve pentagons of Ag_30_ shell and bonded to Ag_12_ shell through σ bond. Each of the twelve ^*t*^BuC≡C^−^ also bonds with the pentagon through π bonds (Fig. 2a). The rest 12 ^*t*^BuC≡C^−^ ligands along with 8 OTf ligands, which amount to 20, coordinated with the 20 triangles of the Ag_30_ shell respectively. It is noteworthy that every ^*t*^BuC≡C^−^ and OTf has the similar μ_3_ coordination mode (Fig. 2b and c). It is also worth noting that although OTf bonding with Ag atoms are commonly seen in Ag(i) complexes or clusters, especially those with low nuclearity,^35,36^ Ag(0) NC protected by OTf has barely been reported. The bond length of the Ag–O bond is 2.415 Å, which is within the common range typical Ag–O bonds.^37,38^

**Fig. 3:**
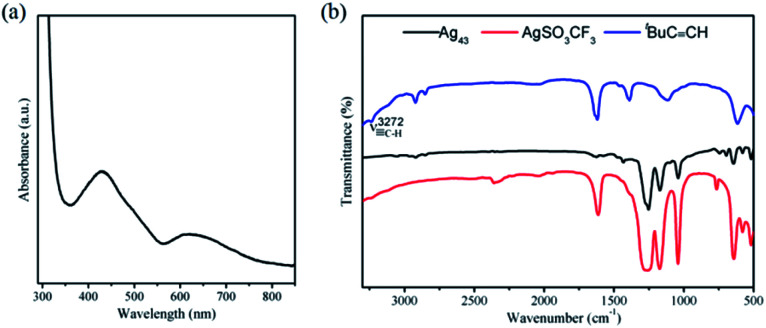
(a) UV-vis absorption spectrum of Ag_43_ in CH_2_Cl_2_ solution. (b) FT-IR spectrum of Ag_43_ (black), ^*t*^BuC≡C^−^ (blue) and AgSO_3_CF_3_ (red).

We investigated the optical properties of Ag_43_ in CH_2_Cl_2_ (Fig. 3). As illustrated in Fig. 3a, the UV-vis absorption spectrum of Ag_43_ shows two absorption features: one major peak at 434 nm and a shoulder at 620 nm. FT-IR spectra of Ag_43_, ^*t*^BuC≡C^−^ and the precursor AgSO_3_CF_3_ are shown in Fig. 3b. The stretching of ≡C–H at 3272 cm^−1^ is no longer present in Ag_43_, indicating the bonding of ^*t*^BuC≡C^−^ with Ag.^39^ The characteristic peaks of SO_3_CF_3_^−^ ranging from 1000 to 1500 cm^−1^, on the other hand, are still prominent in the spectrum of Ag_43_. These information further confirmed both ligands are present on Ag_43_.

**Scheme 1:**
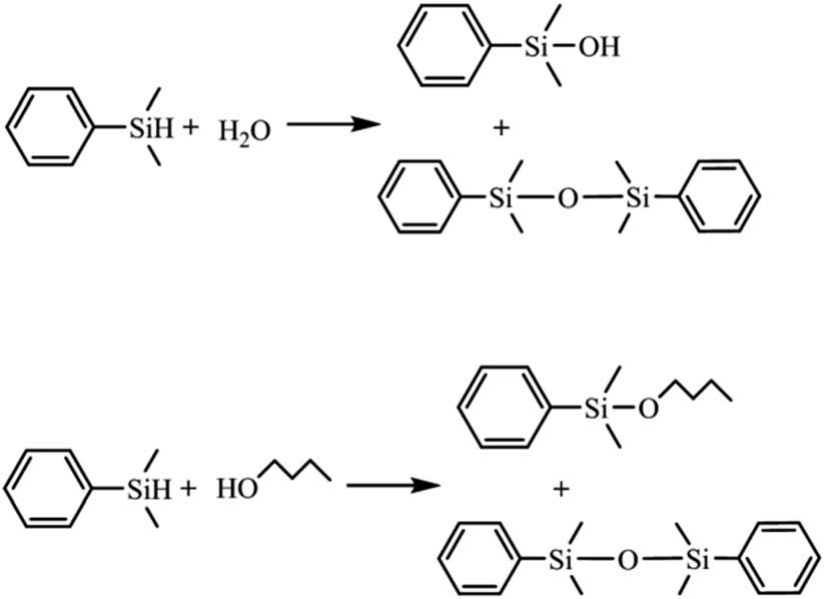
Reaction scheme of silane with H_2_O and 1-butanol. The side reaction is the condensation of two silane molecules.

**Fig. 4:**
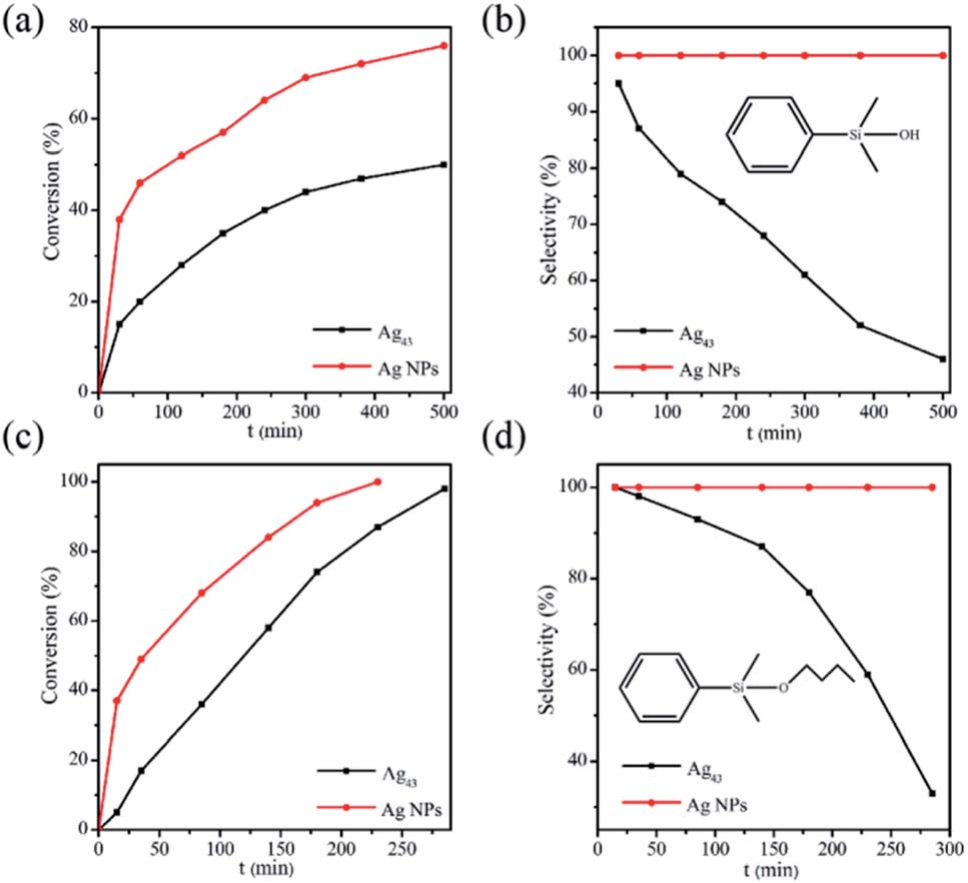
Tracing the reaction of silane with H_2_O catalyzed by Ag_43_ or Ag/PVP. (a) Conversion of silane and (b) the selectivity towards the target whose structure is shown in the figure. (c and d) are those for silane reacting with butanol. Reaction conditions: Ag (0.2 mmol), dimethylphenylsilane (2 mmol), H_2_O or 1-butanol (22 mmol), toluene (2 mL), 30 °C. The byproduct is 1,3-diphenyl-1,1,3,3-tetramethyldisiloxane for both reactions.

The possible influence of the Ag_43_ surface ligands on catalysis was evaluated in the reaction of a sterically demanding substrate dimethylphenylsilane (denoted as silane) with H_2_O or 1-butanol (Scheme 1).^40,41^ As a benchmark, polyvinylpyrrolidone (PVP) stabilized Ag nanoparticles (Ag/PVP) were also fabricated following a reported procedure.^42^ The results are summarized in Fig. 4. We can see that for either H_2_O or alcohol as substrate, the reaction catalyzed by Ag_43_ always showed significantly lower reactivity than Ag/PVP, although the amount of Ag as well as the other reaction conditions were kept the same (Fig. 4a and c). Despite the small size of Ag_43_ (less than 1 nm), the surface ligands still exerted a direct impact on limiting the contact of substrate with Ag. This also influenced the product distribution. For Ag/PVP, full selectivity towards the target was always achieved. Ag_43_, however, afforded a significant amount of 1,3-diphenyl-1,1,3,3-tetramethyldisiloxane (denoted as disiloxane). As the reaction proceeded, the byproduct kept accumulating. For example, in hydrolysis reaction catalyzed by Ag_43_, when half the silane was consumed, the byproduct disiloxane accounted for 54% of the total product (Fig. 4b). The situation was similar in dehydrogenative coupling with 1-butanol (Fig. 4d). Not only the reaction rate was slower for Ag_43_, but disiloxane was also found as a major part of the products. Ag was fairly stable under such reaction condition. The light absorption Ag_43_ after reaction has similar features (Fig. S3†). Generally, the rate-limiting step of this reaction is the activation of alcohol. Thus, given the full occupation of the surface Ag site, this step might be inhibited, which gave silane more chance to react with each other. The exact mechanism, however, still requires further thorough investigation.

## Conclusions

In conclusion, a triflate and *tert*-butylacetylene protected Ag_43_ is synthesized and characterized. For the first time the triflate is shown to function as an effective surface ligand for Ag(0) nanocluster. It bonds with Ag atoms in μ_3_ mode. Although Ag_43_ has a very small size, its catalytic ability is significantly worse than the PVP protected Ag nanoparticles. The surface ligands of Ag_43_ showed strong passivation effect for the reaction of silane with H_2_O or butanol. Such an effect also sharply decreased the selectivity towards the desired target, yielding a significant percentage of disiloxane as byproduct.

## Conflicts of interest

There are no conflicts to declare.

## Supplementary Material

RA-010-D0RA01244K-s001

RA-010-D0RA01244K-s002
