# Supplementary material for: A triflate and alkynyl protected Ag43 nanocluster with a passivated surface
Source: RSC Adv. 2020 May 20;10(33):19397–400. doi: 10.1039/d0ra01244k (PMC9054053; doi:10.1039/d0ra01244k)
Supplement: RA-010-D0RA01244K-s001 [file RA-010-D0RA01244K-s001.pdf]

Electronic Supplementary Material (ESI) for RSC Advances.
This journal is © The Royal Society of Chemistry 2020

Supporting Information

A Triflate and Alkynyl Protected Ag_{43} Nanocluster with Passivated Surface

Ting Li,[a §] Xiaoqin Cui,[b §] Linfeng Liang,[b] Cui Luo,[b] Huan Li,[b]* Xian-Ming Zhang,[ab]*

[a]*School of Chemistry & Material Science, Shanxi Normal University, Linfen, 041000, China*

[b]*Institute of Crystalline Materials, Shanxi University, Taiyuan, 030006, China*

[§] These authors contributed equally to this work.

Corresponding authors: 59584340@sxu.edu.cn (H. Li) xmzhang@sxu.edu.cn (X.-M. Zhang)

Experimental section

1 Materials

Tert-butylacetylene (^{t}BuC≡C$^-$, 98%) and 1,4-bis-(diphenylphosphino)butane(dppb, 97%) were purchased from Shanghai Macklin Biochemical Co., Ltd (China), silver trifluoromethanesulfonate (AgOTf, 99.8%) and sodium borohydride ($NaBH_4$, 98%) were from Energy Chemical (Shanghai, China). All chemicals and solvents were used as received.

2 Instruments

FT-IR spectra were collected on Nicolet iS5 with samples prepared as KBr pellets.

UV-Vis absorption was tested on a domestic spectrophotometer with samples being dispersed in CH_2Cl_2.

Emission spectra were measured on an Edinburgh FLS-920 equipment in solution at room temperature.

X-ray Crystallography. Data collection for $Ag_{43}(^tBuC≡C)_{24}(CF_3SO_3)_8$ was carried on an Agilent Technologies SuperNova Single Crystal Diffractometer using Cu Kα radiation (λ = 1.54178 Å) at 150 K. Absorption corrections were applied by using the program CrysAlis (multi-scan).[1] The structure was solved and refined using Full-matrix least-squares based on F^2 with program SHELXS-97 and SHELXL-97 within OLEX2.[2]

3 Synthesis of $Ag_{43}(^tBuC≡C)_{24}(CF_3SO_3)_8$ (Ag_{43})

Silver trifluoromethanesulfonate (51 mg, 0.2 mmol) was dissolved in ethanol (2 mL), then a CH_2Cl_2 solution (2 mL) containing dppb (1,3-bis(diphenyphosphino)butane, 26 mg, 0.1 mmol) and *tert*-butylacetylene (14 μL, 0.1 mmol) was added under vigorous stirring. A freshly prepared solution of $NaBH_4$ (0.1 mmol in 1 mL of ethanol) was added dropwise after 35 μL triethylamine was introduced. Upon reduction, the solution changed from colorless to light yellow. The reaction was then aged for 12 hours at ambient conditions, during which the color turned dark green. The mixture was evaporated to dryness to give a dark solid, which was dissolved in CH_2Cl_2 (2 mL) again. The solution was centrifuged for 5 min at 12000 r/min. The supernatant solution was subject to the diffusion with n-hexane and diethyl ether (volume ratio, 1:1) to afford black green block crystals after one week.

Characterization

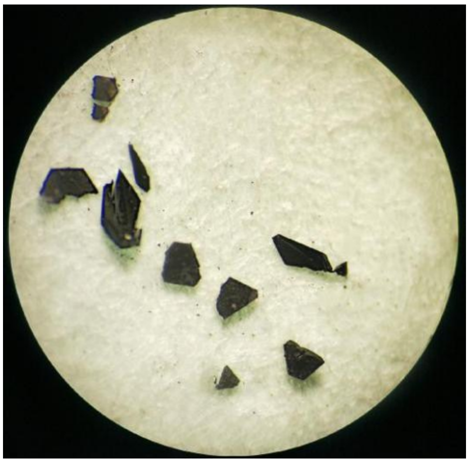

Figure S1. An image of Ag_{43} crystals (black block) under an optical microscope.

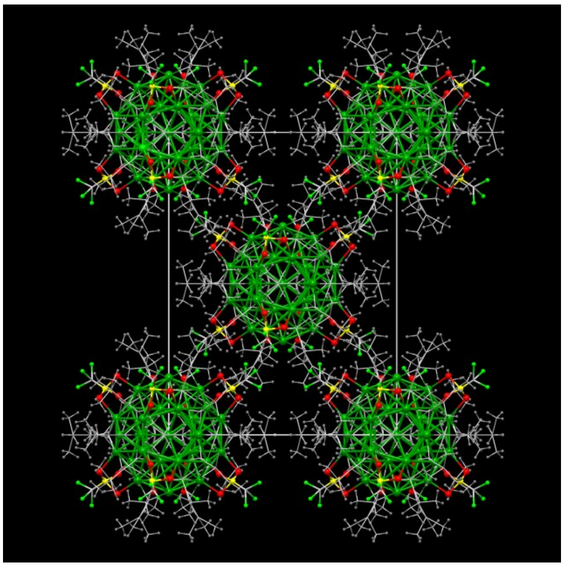

Figure S2. Packing of Ag_{43} in a unit cell. Color legend: Ag, dark green; S, yellow; O, red; F, light green; C, gray.

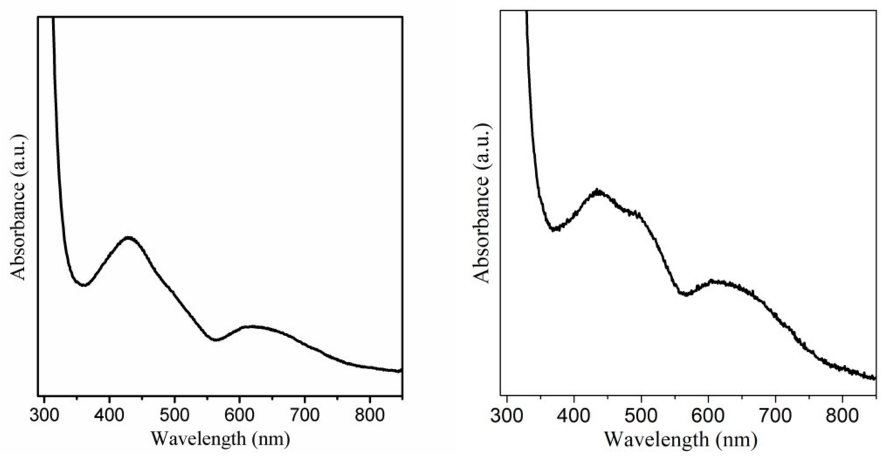

Figure S3. The UV-Vis absorption of Ag_{43} before (left) and after (right) catalyzing the reaction of silane with H_2O.

Table 1. Crystal data and structure refinement for Ag_{43}.

Identification code	$Ag_{43}(^{t}BuC\equiv C)_{24}(CF_3SO_3)_8$
Empirical formula	$C_{154}H_{210}Ag_{43}F_{24}O_{24}S_8$
Formula weight	7796.10
Temperature/K	150
Crystal system	monoclinic
Space group	I2/m
a/Å	19.8306(5)
b/Å	27.6106(10)
c/Å	20.8093(9)
α /°	90
β /°	91.117(3)
γ /°	90
Volume/Å3	11391.7(7)
Z	2
ρ calcg/cm3	2.273
μ /mm-1	30.120
F(000)	7382.0
Crystal size/mm3	0.25 × 0.25 × 0.25
Radiation	CuKα (λ = 1.54184)
2Θ range for data collection/°	8.5 to 159.84
Index ranges	-13 ≤ h ≤ 25, -17 ≤ k ≤ 34, -26 ≤ l ≤ 23
Reflections collected	32054
Independent reflections	12003 [Rint = 0.0503, Rsigma = 0.0504]
Data/restraints/parameters	12003/1015/650
Goodness-of-fit on F2	1.514
Final R indexes [I>=2σ (I)]	R1 = 0.1238, wR2 = 0.3962
Final R indexes [all data]	R1 = 0.1498, wR2 = 0.4187
Largest diff. peak/hole / e Å-3	2.08/-1.87

References

1 CrysAlisPro Version 1.171.35.19. 2011. Agilent Technologies Inc. Santa Clara, CA, USA.

2 Dolomanov, O. V.; Bourhis, L. J.; Gildea, R. J.; Howard, J. A. K.; Puschmann, H. J Appl. Crystallogr. 2009, 42, 339.
